# Sulfasalazine’s potential in managing rheumatoid nodules: Insights from a case report

**DOI:** 10.1097/MD.0000000000039209

**Published:** 2024-08-02

**Authors:** Mohammed Alaswad, Suaad Hamsho, Enas Sultan, Muhammad Al-Ibrahim, Ahmed Merza, Yamen Al-Baroudi

**Affiliations:** a Faculty of Medicine, University of Hama, Hama, Syria; b Rheumatology Department, Faculty of Medicine, Damascus University, Damascus, Syria.

**Keywords:** autoimmune disease, methotrexate, rheumatoid arthritis, rheumatoid nodules, sulfasalazine

## Abstract

**Rationale:**

Rheumatoid arthritis (RA) is a systemic inflammatory disease characterized by joint inflammation and various extra-articular manifestations, including rheumatoid nodules (RNs). This case study aims to explore the effectiveness of alternative treatments for RNs, particularly highlighting the therapeutic potential of sulfasalazine.

**Patient concerns:**

A 52-year-old male with established RA presented with worsening joint pain and firm nodules on his elbows, feet, and fingers.

**Diagnoses:**

The patient fulfilled the diagnostic criteria for RA and was diagnosed with methotrexate-induced RNs based on their temporal association with methotrexate initiation.

**Interventions:**

Methotrexate was discontinued and a combination of leflunomide and sulfasalazine was initiated. Sulfasalazine led to improvement in both joint pain and nodule size. However, due to cost concerns, the patient discontinued sulfasalazine, resulting in a resurgence of both symptoms and nodule enlargement. Reintroduction of methotrexate resulted in significant improvement in joint inflammation, and notably, no new nodules developed at 6 months follow-up.

**Outcomes:**

Sulfasalazine demonstrated efficacy in managing RA nodules, suggesting a potential alternative therapy.

**Lessons:**

The case highlights the complex etiology of nodules in RA and emphasizes the importance of individualized treatment approaches and close monitoring for optimal management.

## 1. Introduction

The autoimmune disease known as rheumatoid arthritis (RA) is a systemic inflammatory disease that mainly affects the joints. It can also cause extra-articular manifestations such as rheumatoid nodules (RNs) and pulmonary vasculitis, in addition to systemic comorbidities.^[1,2]^ The incidence of RA in the whole population is estimated to range from 0.5% to 1%.^[3]^ Generally, women are 2 to 3 times more likely than men to develop RA.^[4]^

RA is characterized by synovial inflammation, the production of autoantibodies such as rheumatoid factor (RF) and anti-citrullinated protein antibody,^[1]^ raised C-reactive protein levels, and an increased erythrocyte sedimentation rate.^[5]^ The first-line therapy for RA is methotrexate (MTX) which is highly effective as monotherapy or when combined with other disease-modifying antirheumatic drugs (DMARDs) or biological treatments.^[6]^

The most prevalent extra-articular manifestation of RA is RNs, which are also seen in other inflammatory and autoimmune diseases.^[7]^ RNs seem to be a sign of more severe disease.^[6]^ Smoking, male gender, elevated levels of serum RF, and anti–cyclic citrullinated peptide antibodies are risk factors for RNs.^[6]^

RNs appearance and progression have long been associated with MTX.^[8]^

In this report, we present a case wherein RNs manifested following the administration of MTX. Subsequent treatment with sulfasalazine resulted in noticeable improvement. However, upon discontinuation of sulfasalazine due to non-adherence, a recurrence of the nodules was observed.

Written informed consent for the publication of this case report was obtained from the patient.

## 2. Case presentation

A 52-year-old man with rheumatoid polyarthritis presented to the rheumatology clinic with complaints of worsening multiple joint pains (including his hands and wrists) and firm nodules on his elbows, feet, and fingers. The patient is a heavy smoker, with a 40-pack-year smoking history.

Three years earlier, he was diagnosed with RA and commenced on MTX (15 mg weekly) as a treatment. During a period of 6 months, the patient developed firm nodules on his feet, elbows, and fingers. MTX-induced nodules were suspected and; therefore, MTX was discontinued, and a combination therapy of leflunomide (20 mg daily) and sulfasalazine (2 g in 4 divided doses per day) was initiated as an alternative therapy. Subsequently, no new nodules appeared during that regimen.

However, 6 months before the latest presentation, sulfasalazine was stopped by the patient due to cost concerns, and he continued with leflunomide alone. As a result, the nodules were markedly enlarged and his symptoms worsened. During these episodes of worsening symptoms and nodules, the patient consistently exhibited an elevated Disease Activity Score-28 score of more than 5.1.

**Figure 1.**
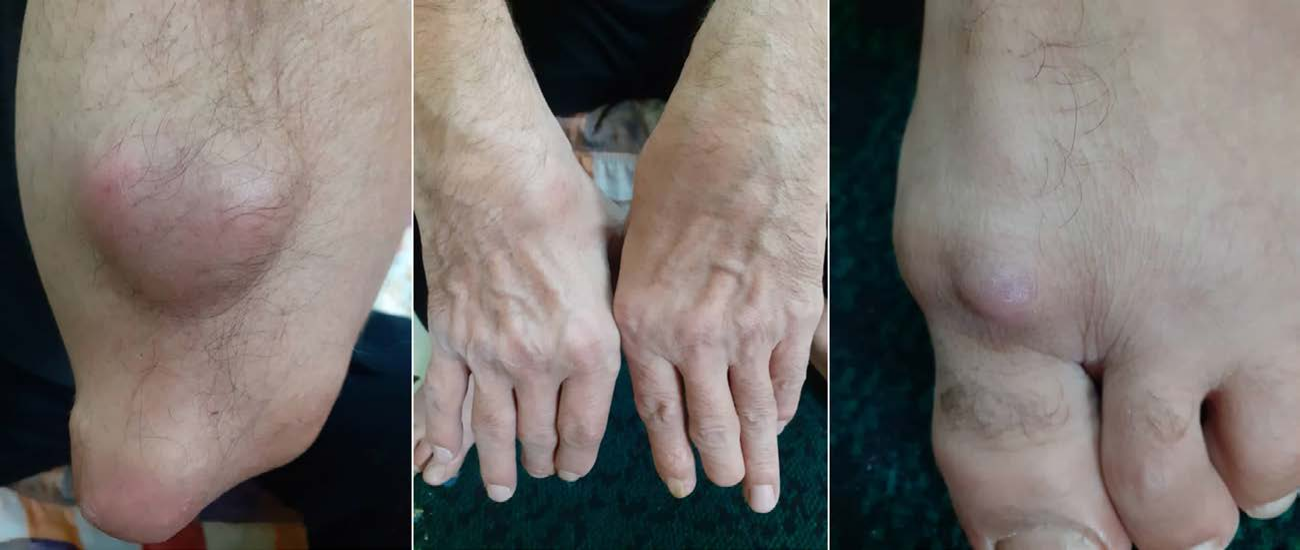
Rheumatoid nodules.

Upon physical examination, the patient exhibited articular swelling and tenderness on palpation. Firm red nodules on his feet, elbow, and fingers were observed (Fig. 1). There was no hoarseness or respiratory symptoms.

**Figure 2.**
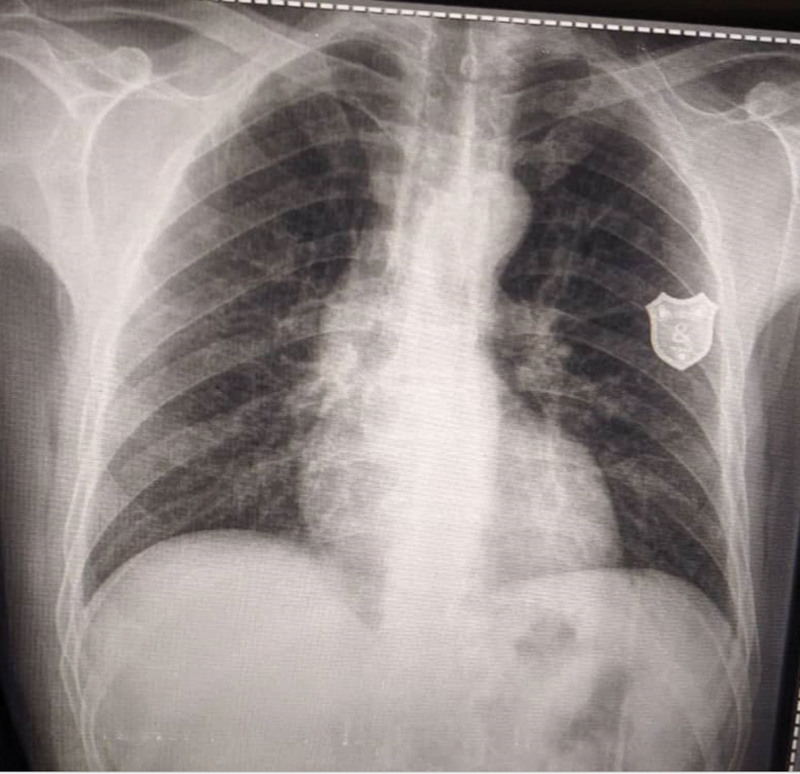
Chest X-rays show normal findings with no evidence of rheumatoid nodules.

Laboratory test results indicated a positive RF and high titer of anti–cyclic citrullinated peptide. Serum uric acid, creatinine, calcium, phosphate, white and red blood cell counts, and liver function tests were all within the normal range (Table 1). Pulmonary function test results were forced expiratory volume in one second/forced vital capacity (71%), forced expiratory volume in one second (73%), and forced vital capacity (82%) and no RNs were evident on the chest X-ray (Fig. 2).

**Table 1:** Laboratory tests

Test	Results	Normal range
White blood cell	7.58	3.5–10·10^3^/µL
Neutrophiles	63	35%–80%
Lymphocytes	32	15%–50%
Monocytes	5	2%–6%
Eosinophils	00	1%–4%
Basophils	00	0%–1%
Red blood cell	4.72	3.5–5.5·10^6^/µL
Hemoglobin	13.2	11.5–16.5 g/dL
Hematocrit	37	35%–55%
Mean corpuscular volume	79	75–100 fl
Mean corpuscular hemoglobin	28	25–35 pg
Mean corpuscular hemoglobin	35	31–38 g/dL
Red cell distribution cell	11.0	11%–16%
Platelet count	378	100–400·10^3^/µL
Erythrocyte sedimentation rate	86	Up to 0.5 mm/h
CRP	109.8	Up to 6 mg/dL
ALT	17	10–60 U/L
AST	21	10–50 U/L
Creatinine	1.11	0.6–1.3 mg/dL
Urea	18	10–40 mg/dL
Anti-CCP	225	0–17 U/mL
RF test	79.4	Up to 30 mg/L

ALT = alanine transaminase, AST = aspartate transaminase, CCP = cyclic citrullinated peptide, CRP = C-reactive protein, RF = rheumatoid factor.

**Figure 3.**
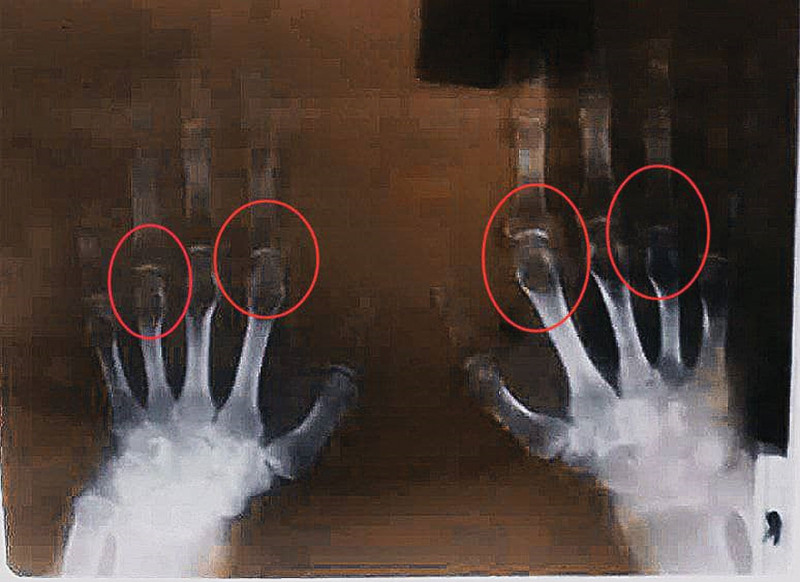
X-rays of the hands reveal bilateral and symmetrical involvement, proximal interphalangeal joint space narrowing, metacarpophalangeal joint space narrowing, and metacarpophalangeal joint osteopenia (red circles).

A radiological examination of both hands revealed bilateral involvement with proximal interphalangeal joint space narrowing, metacarpophalangeal joint space narrowing, and metacarpophalangeal joint osteopenia (Fig. 3).

Due to the exacerbation of the patient’s symptoms and in the absence of absolute contraindications, MTX was reintroduced. This resulted in a significant improvement in his inflammatory condition, and notably, on follow-up 6 months later, no new nodules were observed.

## 3. Discussion

An antifolate metabolite called MTX prevents DNA synthesis, repair, and cellular division. It was originally used in 1951 to treat psoriasis due to its anti-inflammatory and immunomodulatory properties.^[8]^ Recent reports suggest that up to 8% to 10% of RA patients receiving MTX may experience accelerated nodulosis.^[1]^ The regression of the nodules upon stopping MTX therapy supports a cause-and-effect link between the drug’s withdrawal and the growth of nodules.^[9]^

Subcutaneous nodulosis is experienced by up to 35% of RA patients, making it the most common extra-articular symptom. The nodules are frequently linked to elevated RF or anti-citrullinated protein antibody titers, as well as a more aggressive and erosive course of RA. They usually appear on the dorsal surface of the fingers, elbows, or Achilles tendons, though they can also affect the lungs or vocal cords.

There is conflicting evidence linking male gender, cigarette smoking, and a genetic background to the incidence of nodulosis.^[10, 11]^ In this case, the patient is male and a smoker.

The most prominent feature of RA is symmetrical pain and swelling of the hands, wrists, feet, and knees (polyarthritis).^[12]^ One of the typical early signs is hand involvement. Synovitis of metacarpophalangeal, proximal interphalangeal, and wrist joints causes tender swelling on palpation, with early severe motion impairment but no radiologic evidence of bone damage.

In advanced stages of the disease, a spectrum of deformities may manifest, commonly characterized by terms such as “boutonniere deformity” and “swan neck deformity,” denoting the consequential damage and dislocation of finger joints and tendons. The metacarpophalangeal and proximal interphalangeal joints are predisposed to early erosion, marked by the development of small, discrete, pocketed erosions adjacent to the capsular insertion site.^[13]^ Some patients with RA may develop disease manifestations in other organs (sometimes without obvious articular involvement), such as interstitial lung disease, pericarditis, pleural effusion, or bronchiectasis.

Over the past 2 to 3 decades, significant advancements in RA treatment have led to substantial improvements in patient outcomes. These advancements include reduced disease activity, improved functionality, and enhanced quality of life, particularly for patients with moderate to severe RA.^[14]^ The implementation of aggressive therapies involving DMARDs, such as MTX, in combination with biologic agents that selectively target pivotal inflammatory cytokines and pathways, has undoubtedly revolutionized the prognosis for this specific patient population.^[15]^

Nodules often show up without causing symptoms or significant clinical problems and typically do not require specific treatment.^[1]^ Situations that warrant specialized treatment involve pain, entrapment of nerves, or impairment of function. Numerous small-scale studies have shown strong evidence that directly injecting glucocorticoids can greatly reduce the size of RNs.^[5, 14]^ However, the implementation of this procedure is not widespread due to apprehensions regarding infectious complications and the likelihood of nodule reoccurrence. Surgical excision is typically discouraged unless the RN significantly impacts the patient’s quality of life, causing severe pain, nerve compression, skin ulceration, or repeated infections.^[15, 16]^ Nonetheless, there is a scarcity of studies documenting the effectiveness of this therapeutic approach.

Intensive systemic therapy with DMARDs and biologic agents has been shown to effectively reduce joint inflammation and prevent joint damage in RA.^[15, 17]^ While this therapy may also improve various extra-articular manifestations, its effectiveness in specifically treating RNs remains unclear.

The suspension of tumor necrosis factor inhibitor therapy led to a decrease of 30% in pulmonary nodules. Conversely, the use of rituximab, which targets B cells, has demonstrated potential benefits.^[14]^ The absence of nodules prior to MTX administration, followed by their development and subsequent reduction upon stopping the medication, strongly suggests a causal link between MTX and nodulosis. Additionally, some evidence points toward a potential role of genetic factors, particularly HLA-DR4, in susceptibility to MTX-induced nodulosis, although further research is needed in this area.

The optimal treatment approach for MTX nodulosis remains uncertain. While certain studies propose that discontinuing MTX alone is sufficient for nodules to resolve, other researchers argue that hydroxychloroquine may have a beneficial impact on MTX nodulosis even in the absence of MTX cessation.^[13]^ Furthermore, there have been occurrences where nodules have vanished despite the continued use of MTX, leading some experts to suggest that MTX nodulosis should not necessarily be a reason to discontinue MTX in patients who are benefiting from the drug’s therapeutic effects.^[12]^

Several medications have been utilized to address the issue of accelerated nodulosis, including hydroxychloroquine, colchicine, D-penicillamine, and sulfasalazine.^[18]^

According to Englert et al,^[19]^ 4 patients experienced a decline in small RNs upon initiating treatment with sulfasalazine therapy. This coincided with an improvement in synovitis, as well as reductions in erythrocyte sedimentation rate and C-reactive protein levels.^[19]^

In our case, the reduction of nodules upon initiating sulfasalazine shows its potential effectiveness in treating RA nodules. Additionally, the absence of nodular recurrence after reintroducing MTX suggests it may not be responsible for nodules here.

In conclusion, the improvement of RNs with sulfasalazine therapy suggests that this medication may be effective in managing the underlying inflammatory processes associated with RA. Additionally, the fact that the nodules improved with sulfasalazine treatment and may not be directly related to MTX suggests that the etiology of these nodules in this particular case may be more related to the disease process itself rather than a specific medication. However, there are several limitations. As a single case study, the findings may not be generalizable to all patients with RA experiencing RNs. Second, the patient’s discontinuation of sulfasalazine due to cost concerns introduces a variable that complicates the assessment of the medication’s long-term efficacy. Further research with larger patient cohorts and controlled trials is needed to substantiate these findings and clarify the exact mechanisms underlying RA nodulosis and its management. Close monitoring and continued assessment of the patient’s response to treatment will be important to ensure optimal management of their RA and associated symptoms.

## Author contributions

**Conceptualization:** Mohammed Alaswad, Suaad Hamsho.

**Writing—review & editing:** Mohammed Alaswad, Suaad Hamsho.

**Supervision:** Suaad Hamsho.

**Writing—original draft:** Suaad Hamsho, Enas Sultan, Muhammad Al-Ibrahim, Ahmed Merza, Yamen Al-Baroudi.
